# The Transcription Factor MYB37 Positively Regulates Photosynthetic Inhibition and Oxidative Damage in Arabidopsis Leaves Under Salt Stress

**DOI:** 10.3389/fpls.2022.943153

**Published:** 2022-07-12

**Authors:** Yuanyuan Li, Bei Tian, Yue Wang, Jiechen Wang, Hongbo Zhang, Lu Wang, Guangyu Sun, Yongtao Yu, Huihui Zhang

**Affiliations:** ^1^Key Laboratory of Saline-alkali Vegetation Ecology Restoration, Ministry of Education, College of Life Sciences, Northeast Forestry University, Harbin, China; ^2^National Watermelon and Melon Improvement Center, Beijing Academy of Agriculture and Forestry Sciences, Key Laboratory of Biology and Genetic Improvement of Horticultural Crops (North China), Beijing Key Laboratory of Vegetable Germplasm Improvement, Beijing, China

**Keywords:** salt stress, *Arabidopsis thaliana*, transcription factor MYB37, photosynthesis, reactive oxygen species

## Abstract

MYB transcription factors (TFs) mediate plant responses and defenses to biotic and abiotic stresses. The effects of overexpression of *MYB37*, an R2R3 MYB subgroup 14 transcription factors in *Arabidopsis thaliana,* on chlorophyll content, chlorophyll fluorescence parameters, reactive oxygen species (ROS) metabolism, and the contents of osmotic regulatory substances were studied under 100 mM NaCl stress. Compared with the wild type (Col-0), *MYB37* overexpression significantly alleviated the salt stress symptoms in *A. thaliana* plants. Chlorophyll a (Chl *a*) and chlorophyll b (Chl *b*) contents were significantly decreased in OE-1 and OE-2 than in Col-0. Particularly, the Chl *a*/*b* ratio was also higher in OE-1 and OE-2 than in Col-0 under NaCl stress. However, *MYB37* overexpression alleviated the degradation of chlorophyll, especially Chl *a*. Salt stress inhibited the activities of PSII and PSI in Arabidopsis leaves, but did not affect the activity of PSII electron donor side oxygen-evolving complex (OEC). *MYB37* overexpression increased photosynthesis in Arabidopsis by increasing PSII and PSI activities. *MYB37* overexpression also promoted the transfer of electrons from *Q*_A_ to *Q*_B_ on the PSII receptor side of Arabidopsis under NaCl stress. Additionally, *MYB37* overexpression increased Y(II) and Y(NPQ) of Arabidopsis under NaCl stress and decreased Y(NO). These results indicate that *MYB37* overexpression increases PSII activity and regulates the proportion of energy dissipation in Arabidopsis leaves under NaCl stress, thus decreasing the proportion of inactivated reaction centers. Salt stress causes excess electrons and energy in the photosynthetic electron transport chain of Arabidopsis leaves, resulting in the release of reactive oxygen species (ROS), such as superoxide anion and hydrogen peroxide, leading to oxidative damage. Nevertheless, *MYB37* overexpression reduced accumulation of malondialdehyde in Arabidopsis leaves under NaCl stress and alleviated the degree of membrane lipid peroxidation caused by ROS. Salt stress also enhanced the accumulation of soluble sugar (SS) and proline (Pro) in Arabidopsis leaves, thus reducing salt stress damage to plants. Salt stress also degraded soluble protein (SP). Furthermore, the accumulation of osmoregulation substances SS and Pro in OE-1 and OE-2 was not different from that in Col-0 since *MYB37* overexpression in Arabidopsis OE-1, and OE-2 did not significantly affect plants under NaCl stress. However, SP content was significantly higher in OE-1 and OE-2 than in Col-0. These results indicate that *MYB37* overexpression can alleviate the degradation of Arabidopsis proteins under NaCl stress, promote plant growth and improve salt tolerance.

## Introduction

Abiotic stress, especially salt stress, has gradually become the primary factor affecting the survival and distribution of plants due to the change in global climate conditions (Shaheen et al., 2013). Salt stress mainly affects plants in three aspects: (I) Excessive salt in the soil produces osmotic stress. As a result, the water potential becomes lower in soil than in plant root cells, thus inhibiting water absorption (Rana and Mark, 2008). (II) Gradual accumulation of Na^+^ inhibits the absorption of K^+^ in plants, thus affecting some physiological and biochemical reactions that are dependent on K^+^, including enzymatic reactions, protein synthesis, and photosynthesis. Excessive Na^+^ and Cl^−^ also significantly increase intracellular Ca^2+^, resulting in metabolic disorder and even death (Tsugane et al., 1999). (III) Salt stress causes secondary stresses on plants, including oxidative stress and the inhibition of photosynthesis. Excessive reactive oxygen species (ROS) can produce oxidative stress on plants, and damage DNA, enzymes, and biofilm, thus affecting cell structure and metabolism (Dorothea and Ramanjulu, 2005). For instance, salt stress decreases the stability of thylakoid membranes by increasing the rate of chlorophyll degradation in plants, thus hindering the electron transport chain and energy transport of the photosynthetic system and inhibiting photosynthesis (Zhao et al., 2019; Zhang et al., 2020b). Photosynthesis, particularly the photoinhibition of photosystem II (PSII) and photosystem I (PSI), is closely related to ROS production (Che et al., 2018). Excessive ROS disturbs the redox balance in cells, leading to oxidative damage (Jithesh et al., 2006). Therefore, excessive accumulation of ROS in plants under salt stress can significantly affect plant biomass (Klaus and Heribert, 2004). The adaptation of plants to abiotic stress is a complex process involving cell adaptation at the molecular, biochemical and physiological levels (Pushp et al., 2015). The transcriptional machinery associated with stress responses maintains the growth, metabolism, and development of plants through an intricate network of transcription factors (TFs; Agarwal et al., 2013).

Related studies have found that TFs play crucial roles in plant signal regulatory networks. TFs receive the perceived signals and regulate the expression of downstream genes. TFs also act as a node to coordinate the interaction between different signaling pathways. TFs provide complex control mechanisms for plants to manage abiotic and biological stresses, thus regulating developmental processes (Mitsuda and Ohme, 2009). Therefore, the functional study of stress response of transcription factors may provide insights into how plants adapt to severe environments at the molecular level. More than 1,600 TFs have been identified in Arabidopsis (Riechmann et al., 2000; Chen et al., 2006). These TFs can help plants rapidly adapt to changing environments by regulating gene transcription (Zhu, 2002; Kazuo et al., 2003; Chinnusamy et al., 2004). The MYB domain TFs are characterized by a conserved MYB domain with about 52 amino acids involved in DNA binding and are present in all eukaryotes (Yu et al., 2016b). The MYB TF family in Arabidopsis contains 200 genes. It is the largest TF family in Arabidopsis, accounting for 9% of all the TFs in this plant (Riechmann et al., 2000; Søren et al., 2013). Many members of the MYB TF family play a role in tolerance to abiotic stress (Li et al., 2015; Zhang et al., 2020e), regulation of nitrogen absorption and utilization (Liu et al., 2022), and defense responses to pathogens (Mengiste et al., 2003; Liu et al., 2013). The MYB proteins are divided into four subfamilies based on the number of adjacent repeats in the MYB domain (R1-MYB, R2R3-MYB, 3R-MYB, and 4R-MYB; Dubos et al., 2010). The R2R3-MYB family is common in plants (Jiang and Rao, 2020; Wu et al., 2022), with about 126 of these TFs found in Arabidopsis (Chen et al., 2006). Many members of the MYB TF family participate in Arabidopsis response to salt stress (Mengiste et al., 2003; Xie et al., 2010; Cui et al., 2013; Xu et al., 2015; Wang et al., 2016). However, only a few studies have assessed how MYB regulates plant photosynthesis and oxidative damage under salt stress. Previous studies have shown that MYB37, R2R3 MYB subgroup 14 TF in Arabidopsis, affects the phenotypic changes of plant hairy roots by mediating plant hormone signaling pathway. MYB37 also positively regulates plant response to abscisic acid (ABA) and drought stress, thus improving the seed setting rate of Arabidopsis (Dubos et al., 2010; Yu et al., 2016a; Zheng et al., 2020). This study evaluated the effects of *MYB37* overexpression on chlorophyll content, PSII and PSI functions in light reactions, ROS metabolism, and osmotic regulation in Arabidopsis leaves under salt stress. Therefore, this study may provide new insights into how MYB37 alleviates salt stress and provides a theoretical basis for improving the genes related to stress resistance.

## Materials and Methods

### Experimental Materials

The Arabidopsis seeds were disinfected, then sown on MS solid medium. The seeds were vernalized at 4°C for 2 days and cultured in a greenhouse at 21°C, light intensity of 400 μmol·m^−2^·s^−1^, photoperiod of 16/8 h (light/dark), and relative humidity of 60%. *Agrobacterium tumefaciens* containing the recombinant plasmid p MDC85-35 s::MYB37-GFP was used to genetically transform wild-type *A. thaliana* (Col-0) *via* inflorescence infection. The positive transgenic lines were screened based on their resistance to hygromycin. The transgenic plants were verified using PCR and real-time quantitative reverse transcription PCR (qRT-PCR). Genotypic lines (OE-1 and OE-2) with high expression levels of MYB37 in the third generation (T3) homozygous lines were used as the experimental materials. NaCl (100 mmol∙L^−1^) and an equal volume of water were used to irrigate Arabidopsis transgenic lines (OE-1 and OE-2) and Arabidopsis wild type (Col-0) when the seedlings had grown for 4 weeks. A plastic tray was placed under each basin to prevent the loss of salt solution. The solution was poured back into the tray when the matrix was slightly dry. Arabidopsis leaves of each treatment group were randomly sampled on after 7 d irrigation for the following analyses.

### Parameter Measurements and Methods

#### Real-Time PCR Analysis

The 10-day-old seedlings were used to determine the *MYB37* transcript levels in the wild-type Col-0 and the plants overexpressing *MYB37*. Total RNA was extracted from about 100 mg of plant tissue using a Total RNA Rapid Extraction Kit (BioTeke Co., Ltd., Wuxi, China). The total RNA was treated with RNase-free DNaseI (NEB, Ipswich, MA, United States) at 37°C for 1 h to degrade the genomic DNA, then purified using an RNA Purification Kit (BioTeke Co., Ltd.). The total RNA (2 μg) was used to synthesize first-strand cDNA *via* a Roche Transcriptor First Strand cDNA Synthesis Kit (Roche, Basel, Switzerland) and an oligo (dT18) primer. A Bio-Rad Real-Time System CFX96TM C1000 Thermal Cycler (Bio-Rad, Singapore, Singapore) was used for the analysis. ACTIN2/8 genes were amplified and used as the internal control. The cDNA was amplified using SYBR Premix Ex Taq (TaKaRa, Dalian, China) with a DNA Engine Opticon 2 thermal cycler in a 10 μl. All the experiments were repeated at least thrice. The gene-specific primer sequences (5′-3′) were as follows:

MYB37: forward primer: CGACAAGACAAAAGTGAAGCGA.
: reverse primer: TGGCAGCGAAGAGACTAAAAATG.
ACTIN2/8: forward primer: GGTAACATTGTGCTCAGTGGTGG.
: reverse primer: AACGACCTTAATCTTCATGCTGC.

#### Subcellular Localization of MYB37

The roots of 1-week-old MYB37-overexpressing seedlings (OE-2) were immersed in 2 μg/ml 4′,6-diamidino-2-phenylindole (DAPI) solution for 10–15 min for nucleus labeling. The roots were visualized using fluorescence microscopy (EVOS™ FL Auto; Thermo Fisher Scientific, Waltham, MA, United States).

#### Determination of the OJIP Curve and 820 nm Light Reflection Curve (*MR*_820_)

The leaves of Arabidopsis plants were used for a dark adaptation experiment for 30 min using a dark adaptation clip. The OJIP and 820 nm light reflection curves (*MR*_820_) were measured five times using a Hansatech multifunctional plant efficiency instrument (M-PEA; Hansatech Instruments, Ltd., King’s Lynn, United Kingdom) after dark adaptation. The corresponding time points at O, J, I, and P points were 0.01, 2, 30, and 1,000 ms, respectively (represented as *F*_o_, *F*_J_, *F*_I_, and *F*_m_, respectively). Points L and K represent the corresponding points on the curve at 0.15 ms and 0.3 ms, respectively. O-P and O-J were standardized on the OJIP curve. The relative fluorescence intensity (*F*_o_) of the O point was set to 0, while the relative fluorescence intensity (*F*_p_) of the P, J and K points was set to 1 as follows: *V*_O-P_ = (*F*_t_ – *F*_o_) / (*F*_p_ – *F*_o_) and *V*_O-J_ = (*F*_t_ – *F*_o_) / (*F*_J_ – *F*_o_), where *F*_t_ represents the relative fluorescence intensity of each time point. The relative variable fluorescence intensities of the K and J points on the standardization curve were expressed as *V*_K_ and *V*_J_, respectively [*V*_K_ = (*F*_K_ – *F*_o_) / (*F*_J_ – *F*_o_) and *V*_J_ = (*F*_J_ – *F*_o_) / (*F*_P_ – *F*_o_)]. A JIP test analysis was conducted as described by Strasser and Strasser (1995). The PSII maximum photochemical efficiency (*F*_v_/*F*_m_) and photosynthetic performance index were determined based on light absorption (*PI*_ABS_). The slope of the initial section of *MR*_820_ curve (△*I*/*I*_o_, where *I*_o_ and △*I* represent the maximum value and the difference between the maximum value and the minimum value of the reflected signal in 820 nm light reflection curve, respectively) represented the activity of the PSI reaction center (Oukarroum et al., 2018).

#### Determination of Energy Distribution Parameters of the PSII Reaction Center

The maximum fluorescence (*F*_m_) was measured using an FMS-2 pulse modulated fluorometer (Hansatech) after dark adaptation. The steady-state fluorescence (*F*_s_) and maximum steady-state fluorescence (*F*_m_′) were treated at light intensity (PFD) of 1,000 μmol m·^−2^·s^−1^ for light adaptation. The data measured were used to calculate the energy distribution parameters of the PSII reaction center, such as the PSII effective quantum yield Y(II), PSII non-regulated energy dissipation Y(NO), and the PSII regulated energy dissipation yield Y(NPQ) [Y(II) = (*F*_m_′-*F*_s_)/*F*_m_′, Y(NO) = *F*_s_/*F*_m_ and Y(NPQ) = 1-Y(II)-Y(NO)] (Kramer et al., 2004).

#### Determination of Chlorophyll Content

Fresh leaves without main veins were soaked in a 1:1 solution of acetone and ethanol (v/v) to extract the pigments [Chlorophyll a (Chl *a*), Chlorophyll b (Chl *b*), total chlorophyll (Chl *a* + *b*) and chlorophyll a/b (Chl *a*/*b*)] (Porra, 2002).

#### Histochemical Staining of Superoxide Anion (O_2_^−^) and Hydrogen Peroxide (H_2_O_2_)

The superoxide anions (O_2_^−^) and hydrogen peroxide (H_2_O_2_) in fresh leaves were stained using nitro blue tetrazolium chloride (NBT) and 3, 3′-diaminobenzidine tetrahydrochloride (DAB), respectively, as described by Mostofa et al. (2015).

#### Determination of Reactive Oxygen Species (ROS) and Malondialdehyde (MDA) Contents

The rate of production of superoxide anion (O_2_^−^) and the content of hydrogen peroxide (H_2_O_2_) were determined as described by Zhang et al. (2006) and Alexieva et al. (2001). The content of MDA was determined using thiobarbituric acid (TBA) colorimetry (Ernster et al., 1968).

#### Determination of Osmotic Regulatory Substances Content

The contents of soluble sugar (SS), soluble protein (SP), and free proline (Pro) were determined using anthrone colorimetry (Bradford, 1976). Coomassie brilliant blue G-250 staining (Bradford, 1976), acid ninhydrin colorimetry (Bates et al., 1973), respectively.

### Statistical Analysis

Microsoft Excel 2016 (Redmond, WA, United States) and GraphPad Prism 6 software (GraphPad, San Diego, CA, United States) were used for statistical analyses. Data are expressed as mean ± SD. A one-way analysis of variance (ANOVA) and least significant difference (LSD) tests were used to compare the treatments.

## Results and Analysis

### Expression and Subcellular Localization of *MYB37* in Arabidopsis

**Figure 1:**
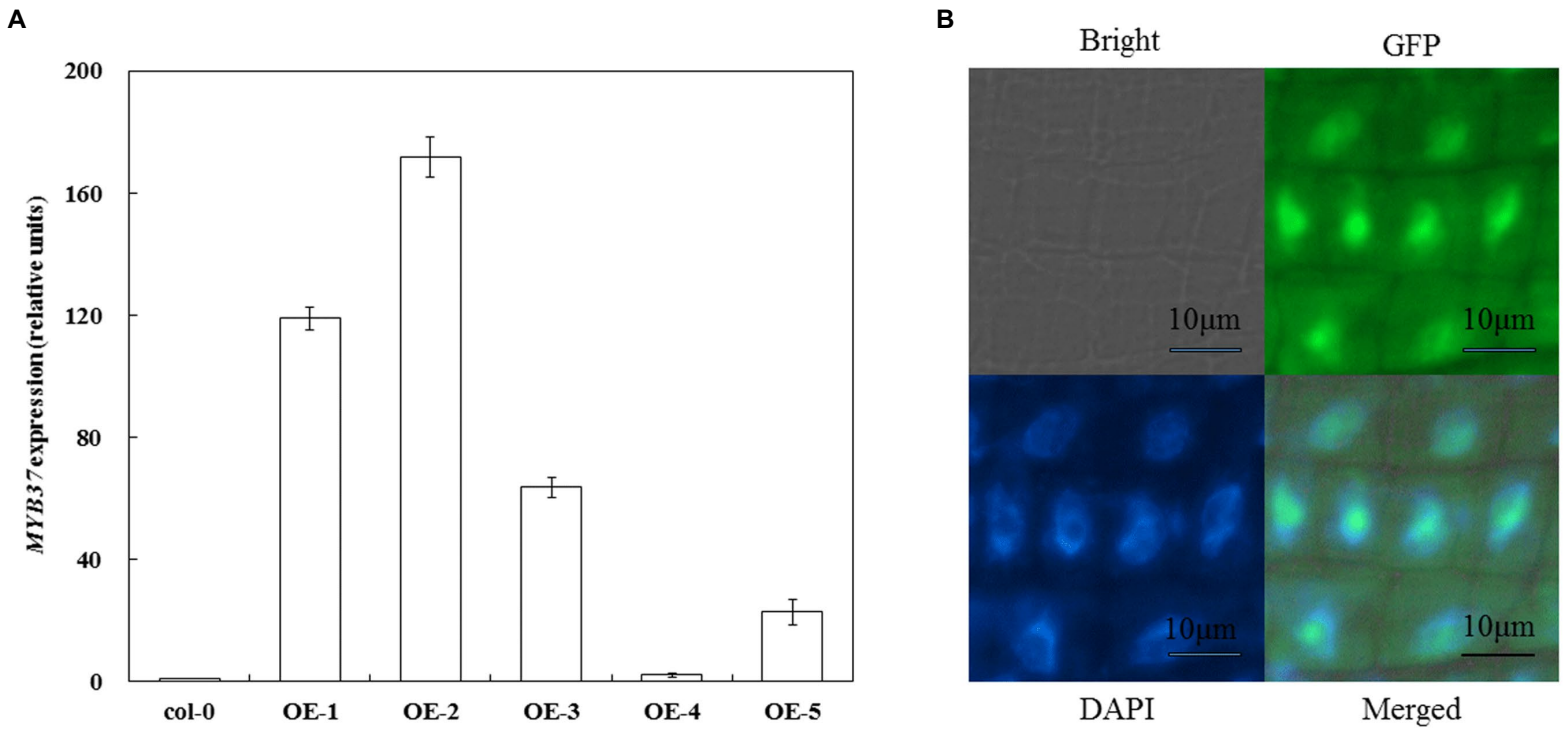
Detection of *MYB37* expression level in Col-0 and overexpressed plants using qRT-PCR **(A)** and subcellular localization of *MYB37* **(B)**.

Arabidopsis overexpressing *MYB37* was obtained *via* transgenic technology to clarify the function of *MYB37* in Arabidopsis under NaCl stress. Real-time quantitative reverse transcription PCR (qRT-PCR) results showed that *MYB37* was significantly expressed in the OE-1 and OE-2 lines than in the other overexpression lines (the expression was more than 100-fold higher than that in Col-0; Figure 1A). Therefore, the OE-1 and OE-2 lines were selected for further functional verification tests. The OE-2 lines with the highest *MYB37* expression were selected to determine the subcellular localization of *MYB37*-GFP fusion protein. High green fluorescent protein (GFP) activity was observed in the nuclear region of the elongation region of Arabidopsis root tips (Figure 1B), indicating that *MYB37* is located in the nucleus.

### Overexpression of the *MYB37* Transcription Factor Improves Salt Stress Tolerance

**Figure 2:**
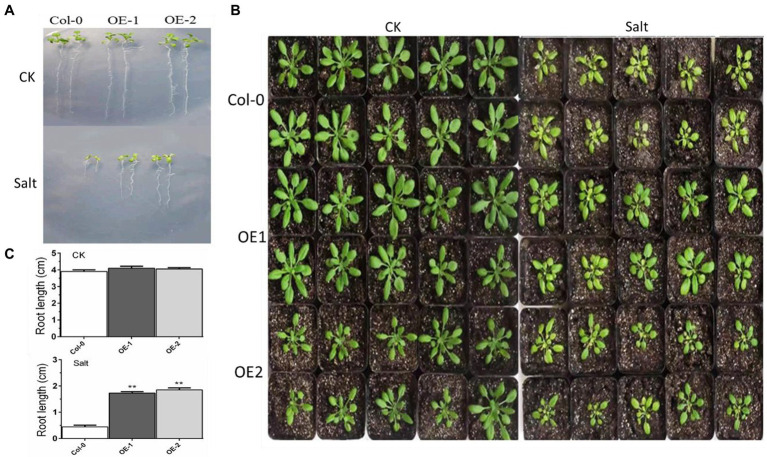
Effects of *MYB37* overexpression on phenotypes of Arabidopsis seedlings **(A)** and 4-week-old adult **(B)** under NaCl stress. Figure 2A shows the phenotype of Arabidopsis seedlings grown on MS medium for 3 days, then transferred to 1/2MS medium with 0 mm or 100 mM NaCl for 7 days. Figure 2B shows the phenotype of Arabidopsis seedlings cultured in the soil after watering with an equal volume of distilled water and 100 mM NaCl solution for 2 weeks. Figure 2C shows statistics of the primary root lengths of the plants as described in **(A)**. Student’s *t*-test was used to compare the primary root lengths of transgenic line with WT plants (with significant differences at **p < 0.01).

The growth of taproots was not significantly different among Col-0, OE-1, and OE-2 Arabidopsis seedlings in normal media. Elongation of the taproots was inhibited in ½ MS media with NaCl. Although there were fewer yellow leaves in OE-1 and OE-2, the root length and number of leaves in the OE-1 and OE-2 plants were significantly higher than Col-0 (Figures 2A,C). The crown width of OE-2 line was slightly lower than that of Col-0 at the 4-week-old adult stage. However, the crown width was not significantly different between OE-1 and Col-0. *MYB37* overexpression significantly relieved the salt damage symptoms of the OE-1 and OE-2 plants aged 4 weeks compared with the Col-0 plants. For instance, *MYB37* overexpression changed the color of the leaves of Col-0 plants from yellow to green (Figure 2B).

### Effects of *MYB37* Overexpression on the Chlorophyll Content in Arabidopsis Leaves Under NaCl Stress

**Figure 3:**
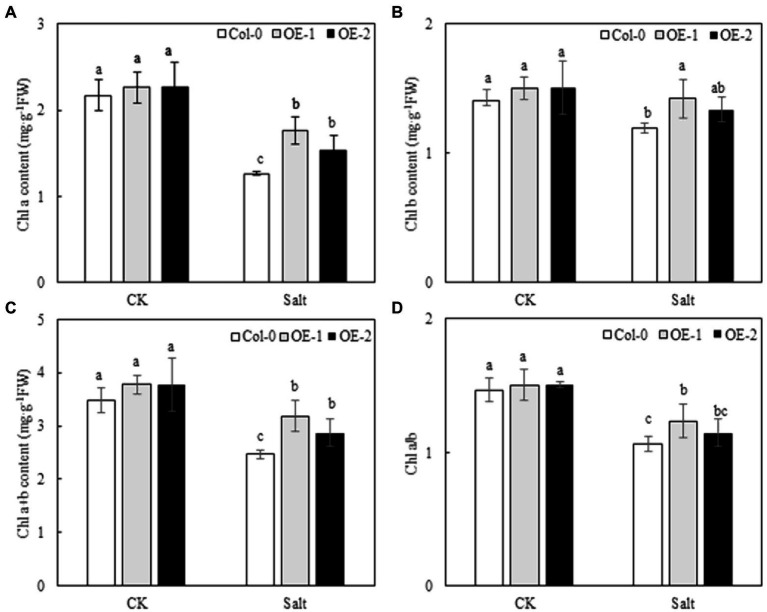
Effects of *MYB37* overexpression on Chl *a* content **(A)**, Chl *b* content **(B)**, Chl *a* + *b* content **(C)**, and Chl *a*/*b* ratio **(D)** in Arabidopsis leaves under NaCl stress. Data are expressed as means ± SE of three replicated experiments (*n* = 3). Different small letters indicate significant differences (*p* < 0.05).

Quantitative analysis showed that the contents of Chl *a*, Chl *b,* and Chl *a* + *b* and the Chl *a*/*b* ratio of Col-0, OE-1, and OE-2 Arabidopsis leaves were not significantly different under normal conditions (Figure 3). NaCl stress degraded chlorophyll and decreased Chl *a*/*b* ratio in Arabidopsis leaves. However, the contents of Chl *a*, Chl *b,* and Chl *a* + *b* were significantly higher in OE-1 and OE-2 lines than in Col-0 under NaCl stress, except for the Chl *b* content, which was not significantly different between the OE-2 lines and Col-0 (Figures 3A–C). Additionally, the Chl *a*/*b* ratio was higher in the OE-1 and OE-2 lines under NaCl stress [20.97 and 7.52% (*p* > 0.05), respectively] than in Col-0 (Figure 3D).

### Effects of *MYB37* Overexpression on the PSII and PSI Activities in Arabidopsis Leaves Under NaCl Stress

**Figure 4:**
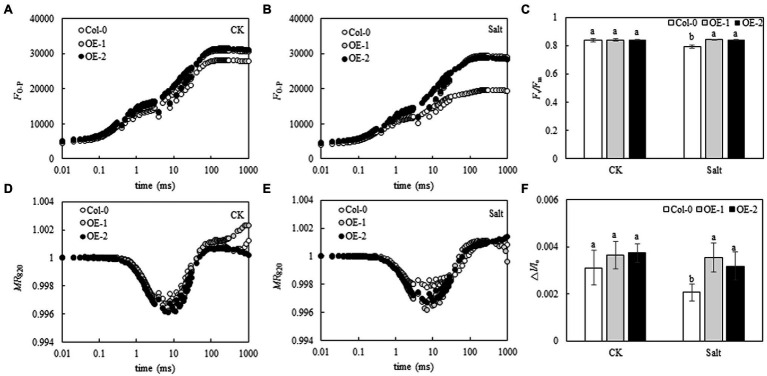
Effects of *MYB37* overexpression on OJIP curve **(A, B)**, MR_820_ curve **(D, E)**, *F*_v_/*F*_m_ **(C)**, and ∆*I*/*I*_o_ **(F)** in Arabidopsis leaves under NaCl stress. Data are expressed as means ± SE of three replicated experiments (*n* = 3). Different small letters indicate significant differences (*p* < 0.05).

Although the relative fluorescence intensity from point J to point P on the OJIP curve was lower in Col-0 Arabidopsis leaves than in OE-1 and OE-2 lines (Figure 4A), *F*_v_/*F*_m_ was not significantly different (Figure 4C). The relative fluorescence intensity of point O slightly changed in Col-0 Arabidopsis leaves. However, the relative fluorescence intensity from point J to point P significantly decreased, and the OJIP curve became relatively flat. The relative fluorescence intensity of OE-1 and OE-2 lines slightly changed (Figure 4B). Compared with OE-1 and OE-2 lines, NaCl stress significantly decreased *F*_v_/*F*_m_ in Col-0 (Figure 4C). Similarly, although the amplitude of *MR*_820_ curve was slightly lower in Col-0 Arabidopsis leaves than in the OE-1 and OE-2 lines under non-stress conditions (Figure 4D), △*I*/*I*_o_ was not significantly different. Moreover, the amplitude of *MR*_820_ curve and △*I*/*I*_o_ of Arabidopsis leaves decreased under NaCl stress (Figure 4E). However, △*I*/*I*_o_ was significantly decreased in Col-0 compared with OE-1 and OE-2 lines under salt stress (Figure 4F).

### Effects of *MYB37* Overexpression on the PSII Receptor Side and Donor Side Electron Transport in Arabidopsis Leaves Under NaCl Stress

**Figure 5:**
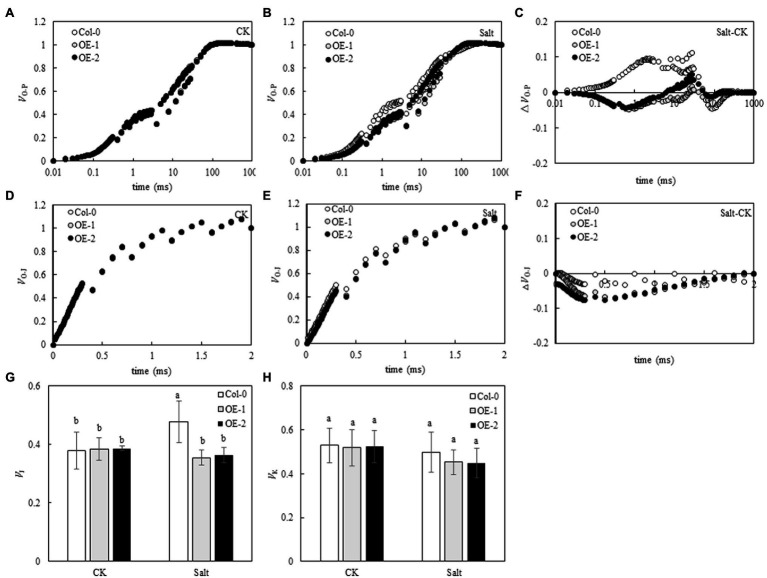
Effects of *MYB37* overexpression on standardized O-P curve **(A, B)**, △*V*_O-P_ curve **(C)**, standardized O-J curve **(D, E)**, △*V*_O-J_ curve **(F)**, *V*_J_ **(G)**, and *V*_K_ **(H)** in Arabidopsis leaves under NaCl stress. Data are expressed as means ± SE of three replicated experiments (*n* = 3). Different small letters indicate significant differences (*p* < 0.05).

O-P normalization of the original OJIP curve showed that the relative fluorescence intensity of each point on the OJIP curve was not significantly different among Col-0 Arabidopsis leaves, OE-1, and OE-2 lines under non-stress conditions (Figure 5A). However, the relative fluorescence intensity of point J on the OJIP curve of Col-0 Arabidopsis leaves substantially changed under NaCl stress compared with OE-1 and OE-2 lines (Figure 5B). The O-P normalized curves of Col-0, OE-1, and OE-2 leaves under NaCl stress were compared with the O-P normalized curves under non-stress. The relative fluorescence intensity at point J on Col-0 curve significantly increased, while it decreased on OE-1 and OE-2 curves (Figure 5C). However, the relative fluorescence intensity was not significant in the quantitative analysis of *V*_J_. Only the *V*_J_ of Col-0 increased by 26.12% under NaCl stress (*p* < 0.05; Figure 5G). Furthermore, the O-J normalized curve of the Col-0, OE-1, and OE-2 leaves were not significantly different under non-stress and NaCl stress conditions (Figures 5D,E). The O-J standardization curves of Col-0, OE-1, and OE-2 Arabidopsis leaves under NaCl stress were compared with those under non-stress conditions. The relative fluorescence intensity at point K of Col-0, OE-1, and OE-2 curves slightly decreased under NaCl stress (Figures 5F,H).

### Effects of *MYB37* Overexpression on the Energy Distribution Parameters of the PSII Reaction Center in Arabidopsis Leaves Under Salt Stress

**Figure 6:**
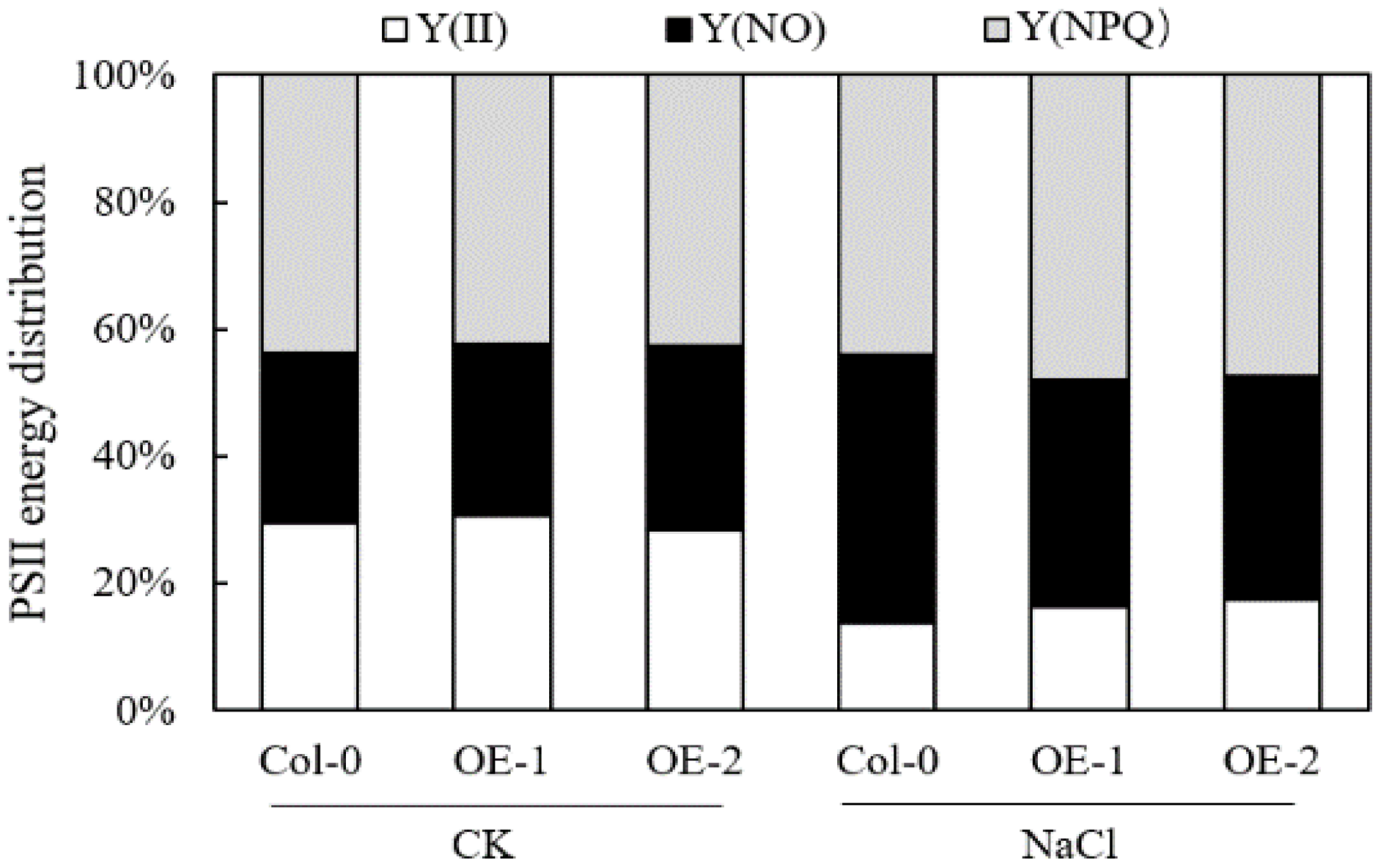
Effects of *MYB37* overexpression on PSII reaction center energy distribution parameters in Arabidopsis leaves under NaCl stress.

Compared with Col-0, MYB37 overexpression did not significantly affect the energy allocation parameters Y(II), Y(NO), and Y (NPQ) of the PSII reaction center in Arabidopsis leaves under non-stress conditions (Figure 6). NaCl stress reduced the proportion of Y(II) in Arabidopsis leaves while it increased the proportion of Y(NO) and Y(NPQ). However, Y(II) and Y(NPQ) were significantly higher in the OE-1, and OE-2 Arabidopsis leaves than in Col-0 under NaCl stress. In contrast, Y(NO) was significantly lower in OE-1, and OE-2 Arabidopsis leaves than in Col-0.

### Effects of *MYB37* Overexpression on the Contents of ROS and MDA in Arabidopsis Leaves Under Salt Stress

**Figure 7:**
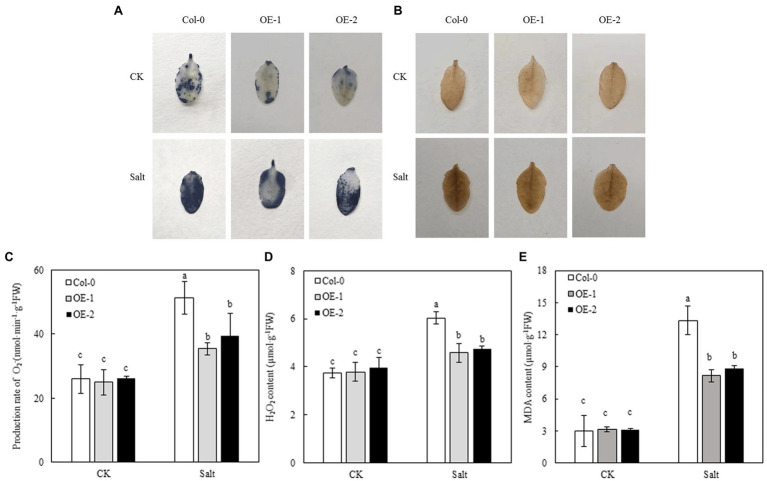
Effects of *MYB37* overexpression on histochemical staining of O_2_^−^ and H_2_O_2_ in fresh leaves **(A, B)**, generation rate of O_2_^−^ **(C)**, H_2_O_2_ content **(D)**, and MDA content **(E)** in Arabidopsis leaves under NaCl stress. Data are expressed as means ± SE of three replicated experiments (*n* = 3). Different small letters indicate significant differences (*p* < 0.05).

NBT and DAB staining were used to detect the accumulation of O_2_^−^ and H_2_O_2_ in Arabidopsis leaves. Less blue sediment accumulated in the OE-1 and OE-2 leaves than in Col-0 under NaCl stress, similar to the accumulation of H_2_O_2_ (yellowish-brown sediment; Figures 7A,B). However, the rate of O_2_^−^/H_2_O_2_ production and MDA contents was not significantly different among Col-0, OE-1, and OE-2 lines under non-stress conditions. In contrast, NaCl stress significantly increased the rate of O_2_^−^ and H_2_O_2_ production and MDA contents of Arabidopsis. Nevertheless, O_2_^−^, H_2_O_2,_ and MDA contents were significantly lower in OE-1 and OE-2 lines than in Col-0 (Figures 7C–E), consistent with the *in situ* staining results of O_2_^−^ and H_2_O_2_.

### Effects of *MYB37* Overexpression on Osmotic Regulatory Substances in Arabidopsis Leaves Under Salt Stress

**Figure 8:**
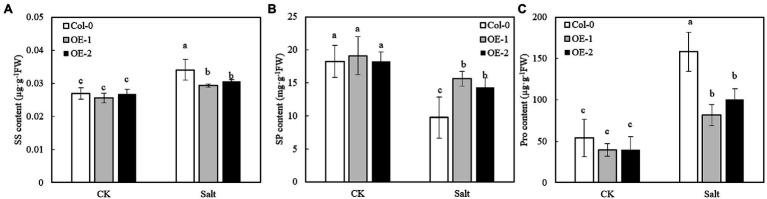
Effects of *MYB37* overexpression on SS content **(A)**, SP content **(B)**, and Pro content **(C)** in Arabidopsis leaves under NaCl stress. Data are expressed as means ± SE of three replicated experiments (*n* = 3). Different small letters indicate significant differences (*p* < 0.05).

The SS, SP, and Pro contents of OE-1, OE-2, and Col-0 were not significantly different under non-stress conditions (Figures 8A–C). However, NaCl stress significantly increased the contents of SS and Pro in Arabidopsis leaves, while it significantly decreased SP contents. Moreover, SS and Pro contents were significantly lower in OE-1 and OE-2 Arabidopsis leaves than in Col-0 under NaCl stress (Figures 8A–C), while SP content was significantly higher than that of Col-0 (Figure 8B).

## Discussion

The ability of transgenic overexpression lines or the loss of function mutants to tolerate abiotic stress is associated with reduced growth or loss of seed productivity (Yu et al., 2016a, 2016b). For instance, although *MYB52*/*MYB96* overexpression confers a dwarf phenotype, while *MYB44*/*MYB61* overexpression reduces seed productivity, the overexpression of these genes improves tolerance to drought or salt stress (Jung et al., 2008; Seo et al., 2009; Park et al., 2011; Romano et al., 2012). In this study, the crown width was slightly lower in Arabidopsis OE-2 line overexpressing *MYB37* than in the wild type. However, MYB37 overexpression in *A. thaliana* maintained green leaves under NaCl stress and significantly alleviated salt stress symptoms. Photosynthesis promotes plant growth and development by providing energy. The photosynthesis of green plants primarily depends on the absorption of light energy by chlorophyll. Therefore, chlorophyll degradation directly reduces the photosynthetic capacity of plants (Zhang et al., 2020d; Siddiqui et al., 2022). Studies have shown that salt stress can inhibit chlorophyll synthesis or degradation in plant leaves (Alaghabary et al., 2005; Ahanger et al., 2019). Herein, salt stress reduced the chlorophyll content of Arabidopsis leaves. Yang et al. (2011) showed that salt stress reduces chlorophyll content in plants through the disruption of Na^+^ ion balance and activity of some proteases. Tulay et al. (2015) also found that salt stress increases the activity of chlorophyllase in *Spergularia marina* (Caryophyllaceae), decreases the content of Mg^2+^ ions, accelerates the degradation of chlorophyll, inhibits the function of pigment protein complex, and the leaves become yellow or even fall off. Herein, *MYB37* overexpression delayed chlorophyll degradation under salt stress and alleviated chlorophyll reduction effect, especially Chl *a*, similar to the results of Bundó et al. (2022). *MYB37* overexpression can exhume or compartmentalize Na^+^ in the cytoplasm into vacuoles, regulate the concentration of Na^+^ in cells and maintain intracellular ion homeostasis by increasing the expression of Na^+^/H^+^ antiporter NHX1 in the vacuolar membrane, thus delaying chlorophyll degradation and enhancing salt tolerance (Zhao et al., 2019). The *MYB* transcription factor also reduces chlorophyll degradation in birch (*Betula* sp.) leaves (Zhou and Li., 2016) and tobacco (*Nicotiana benthamiana*) leaves (Pushp et al., 2015).

Chlorophyll fluorescence can be used to analyze the absorption and utilization of light energy by photosynthesis (Dimitrova et al., 2020; Zhang et al., 2020c). In this study, chlorophyll fluorescence curves (OJIP and *MR*_820_ curves) were used to study the PSII and PSI activities of wild-type Arabidopsis Col-0 and Arabidopsis overexpressing *MYB37* under salt stress. *F*_v_/*F*_m_ and Δ*I*/*I*_o_ are key indexes of photochemical activity in PSII (Giannakoula and Ilias, 2007) and the activity of PSI (Wang et al., 2019), respectively. Herein, salt stress significantly reduced the *F*_v_/*F*_m_ and Δ*I*/*I*_o_ levels in wild-type *A. thaliana* Col-0 compared with normal growth conditions. However, *F*_v_/*F*_m_ and Δ*I*/*I*_o_ levels were not significantly changed in the OE-1 and OE-2 lines of *A. thaliana* overexpressing *MYB37*. Additionally, *F*_v_/*F*_m_ and Δ*I*/*I*_o_ were significantly higher in the OE-1 and OE-2 lines than in Col-0 under salt stress. Salt stress inhibits the activities of PSII and PSI in the leaves of sorghum (*Sorghum bicolor* L.; Zhang et al., 2018b) and halophytic soybean (*Glycine soja*; Yan et al., 2020). Sudhir and Murthy (2004) showed that salt stress inhibits PSII and PSI activities in leaves due to the accumulation of Na^+^ in chloroplasts. In this study, *F*_v_/*F*_m_ and Δ*I*/*I*_o_ were significantly higher in the OE-1 and OE-2 lines than in the wild type, indicating that *MYB37* can improve photosynthesis by increasing the activities of PSII and PSI, thus enhancing salt tolerance. Pushp et al. (2015) also found that *SbMYB15* improves salt tolerance and dehydration in *Salicornia brachia* (highly tolerant to salt) by increasing PSII activity. The electron donor and acceptor sides of the PSII reaction center inhibit photosynthetic electron transport in plants under adverse environmental conditions (Zhang et al., 2020a). *V*_J_ on the OJIP curve can reflect the accumulation of *Q*_A_. The enhancement of *V*_J_ indicates that the electron transfer from *Q*_A_ to *Q*_B_ on the PSII receptor side is blocked (Zhang et al., 2016). The change of *V*_K_ is a specific marker of whether the oxygen-evolving complex (OEC) activity of the PSII electron donor side oxygen release complex is damaged (Zhang et al., 2020d). In this study, salt stress only increased the *V*_J_ value of wild-type Arabidopsis Col-0 curve while slightly changing the *V*_K_ value, indicating that salt stress inhibited the electron transfer from *Q*_A_ to *Q*_B_ on the PSII receptor side of wild-type Arabidopsis leaves. Salt stress did not affect the activity of OEC on the PSII electron donor side. Zhang et al. (2019) also found that salt stress affects OEC activity on the electron donor side of PSII in leaves of mulberry (*Morus alba* L.) after salt and alkali stress treatment. Lu and Vonshak (2002) also found that salt stress reduces the reception of upstream *Q*_A_ electrons by plastoquinone *Q*_B_ (connecting the PSII and PSI reaction centers) in cyanobacteria (*Spirulina platensis*), thus decreasing the electron transfer speed of the entire photosynthetic electron transport chain. Previous studies have also shown that increased Na^+^ content in the cytoplasm and extracellular tissues under salt stress affects the activity of the photosynthetic electron transport chain (Kao et al., 2003). Herein, *MYB37* overexpression alleviated the electron transfer from *Q*_A_ to *Q*_B_ on the PSII receptor side of Arabidopsis under salt stress. Pushp et al. (2015) also proposed that *SbMYB15* could improve the photoprotection mechanism of transgenic lines under salt stress by enhancing electron transfer from the PSII reaction center to the primary quinone receptor. Although stress inhibits the activity of PSII and even leads to the inactivation of PSII response centers, plants adapt to stress by regulating the energy distribution of PSII response centers, such as by increasing energy dissipation (Dimitrova et al., 2020; Sun et al., 2021). In this study, salt stress significantly decreased Y(II) of leaves of *A. thaliana*, while it significantly increased Y(NO) and Y(NPQ). These results indicate that *A. thaliana* adapts to salt stress by increasing its energy dissipation mechanisms. Bashir et al. (2021) showed that salt stress decreases Y(II) in moringa (*Moringa oleifera*), while it increases Y(NO) and Y(NPQ), consistent with this study. Herein, *MYB37* overexpression increased Y(II) and Y(NPQ) of Arabidopsis under salt stress but decreased the Y(NO). These results indicate that *MYB37* overexpression increases the activity of PSII and regulates energy dissipation in Arabidopsis leaves under salt stress, thus decreasing the proportion of inactivated reaction centers.

Photosynthesis inhibition produces excess electrons and energy in the photosynthetic electron transport chain, resulting in an ROS burst and peroxidation damage (Kalaji et al., 2014; Wang et al., 2021b). Wang et al. (2021a) found that salt stress significantly increases O_2_ production rate and the contents of H_2_O_2_ and MDA of alfalfa (*Medicago sativa*) leaves. Zhang et al. (2018a) also found that salt stress increases the rate of O_2_ production and H_2_O_2_ content of mulberry leaves. In this study, salt stress significantly increased the rate of O_2_ production and the contents of H_2_O_2_ and MDA of Arabidopsis leaves. However, ROS and MDA contents were lower in Arabidopsis OE-1 and OE-2 overexpressing *MYB37* than in the wild type under salt stress, consistent with the results of Zhang et al. (2020e) and Huang et al. (2018) in Arabidopsis, tobacco (Pushp et al., 2015) and *Tamarix hispida* (Liu et al., 2021). The overexpression of stress tolerance genes can inhibit membrane damage and significantly reduce the accumulation of ROS and MDA under stress conditions. The content of osmotic regulators changes under osmotic stress, thus improving plant tolerance to abiotic stress (Li et al., 2019). Plants adapt to saline-alkali stress by regulating the accumulation of proline (Pro) and soluble sugar (SS; Ren et al., 2020). Previous studies have shown that Pro and SS regulate plant osmotic balance and improve salt or alkali tolerance (Kanu et al., 2019). Guo et al. (2011, 2017) showed that Pro is significantly accumulated in maize (*Zea mays* L.) under salt stress. Wang et al. (2021a, 2021b) also found that alfalfa leaves can adapt to salt stress by increasing the content of SS and Pro. This experiment also found similar findings described above. In summary, the contents of SS and Pro were significantly increased in Arabidopsis leaves under salt stress, thus reducing salt stress damage to plants. Moreover, *MYB37* overexpression enhanced Arabidopsis OE-1 and OE-2 resistance to salt stress and decreased SS and Pro contents. Pushp et al. (2015) also found similar results in tobacco overexpressing *SbMYB15* under salt stress. Soluble protein (SP) is also a key osmoregulatory substance. Relevant studies have shown that the SP content is substantially accumulated in plant leaves under salt stress (Zhuang et al., 2010; Bai et al., 2013; Hong et al., 2014). In this experiment, salt stress degraded SP in Arabidopsis leaves, similar to Gulen et al. (2006); (strawberry, *Fragaria x ananassa*), Liu et al. (2006); (rice and *Oryza sativa*). However, the accumulation of SP was higher in OE-1 and OE-2 leaves than in the wild type under salt stress, indicating that *MYB37* overexpression promotes protein synthesis of Arabidopsis plant under salt stress and maintains water transport and photosynthetic function of leaves, thus promoting plant growth and salt tolerance (Cernusak et al., 2007).

## Conclusion

Compared with the wild-type (Col-0) Arabidopsis, the overexpression of *MYB37* significantly alleviated the symptoms of salt injury in plants under NaCl stress and alleviated chlorophyll degradation (particularly Chl *a*) under NaCl stress. *MYB37* overexpression also alleviated the photoinhibition of PSII and PSI in Arabidopsis under NaCl stress, particularly by alleviating the electron transfer from *Q*_A_ to *Q*_B_ on the PSII receptor side. *MYB37* overexpression increased the PSII activity, and regulated energy dissipation in Arabidopsis leaves under salt stress, thus decreasing the proportion of inactivated reaction centers. *MYB37* overexpression also reduced the accumulation of ROS and MDA in Arabidopsis leaves under NaCl stress, thus alleviating the oxidative damage. In addition, *MYB37* overexpression alleviated SP degradation in Arabidopsis leaves under salt stress. However, *MYB37* overexpression did not enhance plant adaption to NaCl stress by accumulating SS and Pro due to the strong resistance to NaCl stress.

## Data Availability Statement

The original contributions presented in the study are included in the article/supplementary material, further inquiries can be directed to the corresponding authors.

## Author Contributions

HZ and YY conceived and designed the experiments. YL, BT, and YW wrote the manuscript and prepared the figures and tables. All the authors performed the experiments and analyzed the data. YL, HZ, and YY reviewed drafts of the paper. Sun Guangyu supervised the work. All authors contributed to the article and approved the submitted version.

## Funding

This research was supported by the Project Funded by China Postdoctoral Science Foundation (2022M710023), Fundamental Research Funds for the Central Universities (2572022BD01), National Natural Science Foundation of China (NSFC grant no. 31900228, 31901088, 52004067).

## Conflict of Interest

The authors declare that the research was conducted in the absence of any commercial or financial relationships that could be construed as a potential conflict of interest.

## Publisher’s Note

All claims expressed in this article are solely those of the authors and do not necessarily represent those of their affiliated organizations, or those of the publisher, the editors and the reviewers. Any product that may be evaluated in this article, or claim that may be made by its manufacturer, is not guaranteed or endorsed by the publisher.

## References

[ref1] Agarwal P. K. Shukla P. S. Gupta K. Jha B. (2013). Bioengineering for salinity tolerance in plants: state of the art. Mol. Biotechnol. 54, 102–123. doi: 10.1007/s12033-012-9538-3, PMID: 22539206

[ref2] Ahanger M. A. Qin C. Begum N. Maodong Q. Dong X. X. El-Esawi M.. (2019). Nitrogen availability prevents oxidative effects of salinity on wheat growth and photosynthesis by up-regulating the antioxidants and osmolytes metabolism, and secondary metabolite accumulation. BMC Plant Biol. 19:479. doi: 10.1186/s12870-019-2085-3, PMID: 31703619 PMC6839093

[ref3] Alaghabary K. Zhu Z. Shi Q. (2005). Influence of silicon supply on chlorophyll content, chlorophyll fluorescence, and antioxidative enzyme activities in tomato plants under salt stress. J. Plant Nutr. 27, 2101–2115. doi: 10.1081/PLN-200034641

[ref4] Alexieva V. Sergiev I. Mapelli S. Karanov E. (2001). The effect of drought and ultraviolet radiation on growth and stress markers in pea and wheat. Plant Cell Environ. 24, 1337–1344. doi: 10.1046/j.1365-3040.2001.00778.x

[ref5] Bai Q. Yang R. Zhang L. Gu Z. (2013). Salt stress induces accumulation of γ–aminobutyric acid in germinated foxtail millet (*Setaria italica* L.). Cereal Chem. 90, 145–149. doi: 10.1094/CCHEM-06-12-0071-R

[ref601] Bashir S. Amir M. Bashir F. Javed M. Hussain A. Fatima S.. (2021). Structural and functional stability of photosystem-II in *Moringa oleifera* under salt stress. 5, 676–682.

[ref6] Bates L. B. Waldren R. P. Teare I. D. (1973). Rapid determination of free proline for water-stress studies. Plant Soil 39, 205–207. doi: 10.1007/BF00018060

[ref7] Bradford M. M. (1976). A rapid and sensitive method for the quantitation of microgram quantities of protein utilizing the principle of protein-dye binding. Anal. Biochem. 72, 248–254. doi: 10.1016/0003-2697(76)90527-3, PMID: 942051

[ref8] Bundó M. Martín C. H. Pesenti M. Gómez A. J. Castillo L. Frouin J.. (2022). Integrative approach for precise genotyping and transcriptomics of salt tolerant introgression Rice lines. Front. Plant Sci. 12:797141. doi: 10.3389/fpls.2021.797141, PMID: 35126422 PMC8813771

[ref9] Cernusak L. A. Aranda J. Marshall J. D. Winter K. (2007). Large variation in whole-plant water-use efficiency among tropical tree species. New Phytol. 173, 294–305. doi: 10.1111/j.1469-8137.2006.01913.x, PMID: 17204076

[ref10] Che X. Ding R. Li Y. Zhang Z. Gao H. Wang W. (2018). Mechanism of long-term toxicity of CuO NPs to microalgae. Nanotoxicology 12, 923–939. doi: 10.1080/17435390.2018.1498928 30182775

[ref11] Chen Y. Yang X. He K. Liu M. Li J. Gao Z.. (2006). The MYB transcription factor superfamily of Arabidopsis: expression analysis and phylogenetic comparison with the rice MYB family. Plant Mol. Biol. 60, 107–124. doi: 10.1007/s11103-005-2910-y 16463103

[ref12] Chinnusamy V. Schumaker K. Zhu J. K. (2004). Molecular genetic perspectives on cross-talk and specificity in abiotic stress signalling in plants. J. Exp. Bot. 55, 225–236. doi: 10.1093/jxb/erh005, PMID: 14673035

[ref13] Cui M. H. Yoo K. S. Hyoung S. Nguyen H. T. K. Kim Y. Y. Kim H. J.. (2013). An Arabidopsis R2R3-MYB transcription factor, AtMYB20, negatively regulates type 2C serine/threonine protein phosphatases to enhance salt tolerance. FEBS Lett. 587, 1773–1778. doi: 10.1016/j.febslet.2013.04.028, PMID: 23660402

[ref14] Dimitrova S. Paunov M. Pavlova B. Dankov K. Kouzmanova M. Velikova V.. (2020). Photosynthetic efficiency of two *Platanus orientalis* L. ecotypes exposed to moderately high temperature: JIP-test analysis. Photosynthetica 58, 657–670. doi: 10.32615/ps.2020.012

[ref15] Dorothea B. Ramanjulu S. (2005). Drought and salt tolerance in plants. Crit. Rev. Plant Sci. 24, 23–58. doi: 10.1080/07352680590910410

[ref16] Dubos C. Stracke R. Grotewold E. Weisshaar B. Martin C. Lepiniec L. (2010). MYB transcription factors in Arabidopsis. Trends Plant Sci. 15, 573–581. doi: 10.1016/j.tplants.2010.06.005 20674465

[ref17] Ernster L. Nordenbrand K. Orrenius S. Das M. L. (1968). Microsomal lipid peroxidation. Biol. Chem. 349, 1604–1605. 4393646

[ref18] Giannakoula A. Ilias I. F. (2007). Chlorophyll fluorescence and photosystem II activity of tomato leaves as affected by irradiance and prohexadione-calcium. Proc. WSEAS Int. Conf. Renew. Energy Sources 91, 49–56.

[ref19] Gulen H. Turhan E. Eris A. (2006). Changes in peroxidase activities and soluble proteins in strawberry varieties under salt-stress. Acta Physiol. Plant. 28, 109–116. doi: 10.1007/s11738-006-0037-7

[ref20] Guo R. Shi L. X. Yan C. Zhong X. Gu F. X. Liu Q.. (2017). Ionomic and metabolic responses to neutral salt or alkaline salt stresses in maize (*Zea mays* L.) seedlings. BMC Plant Biol. 17:41. doi: 10.1186/s12870-017-0994-6, PMID: 28187710 PMC5301417

[ref21] Guo R. Zhou J. Hao W. P. Gong D. Z. Zhong X. L. Gu F. X.. (2011). Germination, growth, photosynthesis and ionic balance in *Setaria viridis* seedlings subjected to saline and alkaline stress. Can. J. Plant Sci. 91, 1077–1088. doi: 10.4141/cjps10167

[ref22] Hong C. T. Guo H. P. Fang J. Ren W. Wang T. F. Ji M. C.. (2014). Physiological and biochemical responses of *miscanthus sacchariflorus* to salt stress. Adv. Mater. Res. 1051, 333–340. doi: 10.4028/www.scientific.net/AMR.1051.333

[ref23] Huang Y. Zhao H. Gao F. Yao P. Deng R. Li C.. (2018). A R2R3-MYB transcription factor gene, FtMYB13, from Tartary buckwheat improves salt/drought tolerance in Arabidopsis. Plant Physiol. Biochem. 132, 238–248. doi: 10.1016/j.plaphy.2018.09.012, PMID: 30227384

[ref24] Jiang C. K. Rao G. Y. (2020). Insights into the diversification and evolution of R2R3-MYB transcription factors in plants. Plant Physiol. 183, 637–655. doi: 10.1104/pp.19.01082, PMID: 32291329 PMC7271803

[ref25] Jithesh M. N. Prashanth S. R. Sivaprakash K. R. Parida A. K. (2006). Antioxidative response mechanisms in halophytes: their role in stress defence. J. Genet. 85, 237–254. doi: 10.1007/BF02935340, PMID: 17406103

[ref26] Jung C. Seo J. S. Han S. W. Koo Y. J. Kim C. H. Song S. I.. (2008). Overexpression of AtMYB44 enhances stomatal closure to confer abiotic stress tolerance in transgenic Arabidopsis. Plant Physiol. 146, 623–635. doi: 10.1104/pp.107.110981, PMID: 18162593 PMC2245844

[ref27] Kalaji H. M. Oukarroum A. Alexandrov V. Kouzmanova M. Brestic M. Zivcak M.. (2014). Identification of nutrient deficiency in maize and tomato plants by in vivo chlorophyll a fluorescence measurement. Plant Physiol. Biochem. 81, 16–25. doi: 10.1016/j.plaphy.2014.03.029, PMID: 24811616

[ref28] Kanu A. S. Ashraf U. Mo Z. Sabir S. U. R. Baggie I. Charley C. S.. (2019). Calcium amendment improved the performance of fragrant rice and reduced metal uptake under cadmium toxicity. Environ. Sci. Pollut. Res. 26, 24748–24757. doi: 10.1007/s11356-019-05779-7, PMID: 31240656

[ref29] Kao W. Y. Tsai T. T. Shih C. N. (2003). Photosynthetic gas exchange and chlorophyll a fluorescence of three wild soybean species in response to NaCl treatments. Photosynthetica 41, 415–419. doi: 10.1023/B:PHOT.0000015466.22288.23

[ref30] Kazuo S. Kazuko Y. S. Motoaki S. (2003). Regulatory network of gene expression in the drought and cold stress responses. Curr. Opin. Plant Biol. 6, 410–417. doi: 10.1016/S1369-5266(03)00092-X 12972040

[ref31] Klaus A. Heribert H. (2004). Reactive oxygen species: metabolism, oxidative stress, and signal transduction. Annu. Rev. Plant Biol. 55, 373–399. doi: 10.1146/annurev.arplant.55.031903.141701 15377225

[ref32] Kramer D. M. Johnson G. Kiirats O. Edwards G. E. (2004). New fluorescence parameters for the determination of Q_A_ redox state and excitation energy fluxes. Photosynth. Res. 79, 209–218. doi: 10.1023/B:PRES.0000015391.99477.0d, PMID: 16228395

[ref33] Li Z. Fu X. Tian Y. Xu J. Gao J. Wang B.. (2019). Overexpression of a trypanothione synthetase gene from Trypanosoma cruzi, TcTrys, confers enhanced tolerance to multiple abiotic stresses in rice. Gene 710, 279–290. doi: 10.1016/j.gene.2019.06.018, PMID: 31200083

[ref34] Li C. Ng C. K. Y. Fan L. M. (2015). MYB transcription factors, active players in abiotic stress signaling. Environ. Exp. Bot. 114, 80–91. doi: 10.1016/j.envexpbot.2014.06.014

[ref35] Liu Z. Y. Li X. P. Zhang T. Q. Wang Y. Y. Wang C. Gao C. Q. (2021). Overexpression of ThMYB8 mediates salt stress tolerance by directly activating stress-responsive gene expression. Plant Sci. 302:110668. doi: 10.1016/j.plantsci.2020.110668, PMID: 33288032

[ref36] Liu X. Liu H. F. Li H. L. An X. H. Song L. Q. You C. X.. (2022). MdMYB10 affects nitrogen uptake and reallocation by regulating the nitrate transporter MdNRT2.4–1 in the red flesh apple. Horticulture Research 9, 1–13. doi: 10.1093/hr/uhac016 PMC9016894 35184189

[ref37] Liu K. Xu S. Xuan W. Ling T. F. Cao Z. Huang B.. (2006). Carbon monoxide counteracts the inhibition of seed germination and alleviates oxidative damage caused by salt stress in *Oryza sativa*. Plant Sci. 172, 544–555. doi: 10.1016/j.plantsci.2006.11.007

[ref38] Liu X. Yang L. Zhou X. Zhou M. Lu Y. Ma L.. (2013). Transgenic wheat expressing *Thinopyrum intermedium* MYB transcription factor TiMYB2R-1 shows enhanced resistance to the take-all disease. J. Exp. Bot. 64, 2243–2253. doi: 10.1093/jxb/ert084, PMID: 23547108 PMC3654416

[ref39] Lu C. Vonshak A. (2002). Effects of salinity on photosystem II function in cyanobacterial Spirulina platensis cells. Physiol Plant. Physiologia Plantarum 114, 405–413. doi: 10.1034/j.1399-3054.2002.1140310.x 12060263

[ref40] Mengiste T. Chen X. Salmeron J. Dietrich R. (2003). The BOTRYTIS SUSCEPTIBLE1 gene encodes an R2R3MYB transcription factor protein that is required for biotic and abiotic stress responses in Arabidopsis. Plant Cell 15, 2551–2565. doi: 10.1105/tpc.014167, PMID: 14555693 PMC280560

[ref41] Mitsuda N. Ohme T. M. (2009). Functional analysis of transcription factors in Arabidopsis. Plant Cell Physiol. 50, 1232–1248. doi: 10.1093/pcp/pcp075, PMID: 19478073 PMC2709548

[ref42] Mostofa M. G. Rahman A. Ansary M. M. U. Watanabe A. Fujita M. Tran L. S. P. (2015). Hydrogen sulfide modulates cadmium-induced physiological and biochemical responses to alleviate cadmium toxicity in rice. Sci. Rep. 5:14078. doi: 10.1038/srep14078, PMID: 26361343 PMC4566128

[ref43] Oukarroum A. Goltsev V. Strasser R. J. (2018). Temperature effects on pea plants probed by simultaneous measurements of the kinetics of prompt fluorescence, delayed fluorescence and modulated 820 nm reflection. PLoS One 8:e59433. doi: 10.1371/journal.pone.0059433 PMC3602342 23527194

[ref44] Park M. Y. Kang J. Y. Kim S. Y. (2011). Overexpression of AtMYB52 confers ABA hypersensitivity and drought tolerance. Mol. Cells 31, 447–454. doi: 10.1007/s10059-011-0300-7, PMID: 21399993 PMC3887605

[ref45] Porra R. J. (2002). The chequered history of the development and use of simultaneous equations for the accurate determination of chlorophylls a and b. Photosynth. Res. 73, 149–156. doi: 10.1023/A:1020470224740 16245116

[ref46] Pushp S. S. Kapil G. Parinita A. Bhavanath J. Pradeep K. A. (2015). Overexpression of a novel SbMYB15 from Salicornia brachiataconfers salinity and dehydration tolerance by reduced oxidative damage and improved photosynthesis in transgenic tobacco. Planta 242, 1291–1308. doi: 10.1007/s00425-015-2366-5 26202734

[ref47] Rana M. Mark T. (2008). Mechanisms of salinity tolerance. Annu. Rev. Plant Biol. 59, 651–681. doi: 10.1146/annurev.arplant.59.032607.092911 18444910

[ref48] Ren Y. Wang W. He J. Zhang L. Wei Y. Yang M. (2020). Nitric oxide alleviates salt stress in seed germination and early seedling growth of pakchoi (*Brassica chinensis* L.) by enhancing physiological and biochemical parameters. Ecotoxicol. Environ. Saf. 187:109785. doi: 10.1016/j.ecoenv.2019.109785, PMID: 31644988

[ref49] Riechmann J. L. Heard J. Martin G. Reuber L. Jiang C. Keddie J.. (2000). Arabidopsis transcription factors: genome-wide comparative analysis among eukaryotes. Science 290, 2105–2110. doi: 10.1126/science.290.5499.2105 11118137

[ref50] Romano J. M. Dubos C. Prouse M. B. Wilkins O. Hong H. Poole M.. (2012). AtMYB61, an R2R3-MYB transcription factor, functions as a pleiotropic regulator via a small gene network. New Phytol. 195, 774–786. doi: 10.1111/j.1469-8137.2012.04201.x, PMID: 22708996

[ref52] Seo P. J. Xiang F. Qiao M. Park J. Y. Lee Y. N. Kim S. G.. (2009). The MYB96 transcription factor mediates abscisic acid signaling during drought stress response in Arabidopsis. Plant Physiol. 151, 275–289. doi: 10.1104/pp.109.144220, PMID: 19625633 PMC2735973

[ref53] Shaheen S. Naseer S. Ashraf M. Akram N. A. (2013). Salt stress affects water relations, photosynthesis, and oxidative defense mechanisms in *Solanum melongena* L. J. Plant Interact. 8, 85–96. doi: 10.1080/17429145.2012.718376

[ref55] Siddiqui M. H. Mukherjee S. Al-Munqedhi B. M. A. Kumar R. Kalaji H. M. (2022). Salicylic acid and silicon impart resilience to lanthanum toxicity in *Brassica juncea* L. seedlings. Plant Growth Regul. 1–14. doi: 10.1007/s10725-021-00787-5

[ref56] Søren L. Charlotte O. Michael J. Karen S. (2013). Structure, function and networks of transcription factors involved in abiotic stress responses. Int. J. Mol. Sci. 14, 5842–5878. doi: 10.3390/ijms14035842 23485989 PMC3634440

[ref57] Strasser B Strasser R. (1995). Measuring fast fluorescence transients to address environmental questions: the JIP-test. Photosynthesis: from light to biosphere, Volume V, Proceedings of the Xth International Photosynthesis Congress, Montpellier, France, 20–25 August 1995, 977–980.

[ref58] Sudhir P. Murthy S. D. S. (2004). Effects of salt stress on basic processes of photosynthesis. Photosynthetica 42, 481–486. doi: 10.1007/S11099-005-0001-6

[ref59] Sun H. W. Zhang H. B. Xu Z. S. Wang Y. Liu X. Q. Li Y. Y.. (2021). TMT-based quantitative proteomic analysis of the effects of *Pseudomonas syringae* pv. *Tabaci* (*Pst*) infection on photosynthetic function and the response of the MAPK signaling pathway in tobacco leaves. Plant Physiol. Biochem. 166, 657–667. doi: 10.1016/j.plaphy.2021.06.049, PMID: 34214776

[ref60] Tsugane K. Kobayashi K. Niwa Y. Ohba Y. Wada K. Kobayashi H. (1999). A recessive Arabidopsis mutant that grows photoautotrophically under salt stress shows enhanced active oxygen detoxification. Plant Cell 11, 1195–1206. doi: 10.1105/tpc.11.7.1195, PMID: 10402422 PMC144266

[ref61] Tulay A. A. Adnan A. Erkan Y. (2015). Anatomical adaptations to salinity in *Spergularia marina* (*Caryophyllaceae*) from Turkey. Proc. Natl. Acad. Sci. India 85, 625–634. doi: 10.1007/s40011-014-0386-8

[ref62] Wang Y. Jin W. W. Che Y. H. Huang D. Wang J. C. Zhao M. C.. (2019). Atmospheric nitrogen dioxide improves photosynthesis in mulberry leaves via effective utilization of excess absorbed light energy. Forests 10:312. doi: 10.3390/f10040312

[ref63] Wang F. Kong W. Wong G. Fu L. Peng R. Li Z.. (2016). AtMYB12 regulates flavonoids accumulation and abiotic stress tolerance in transgenic *Arabidopsis thaliana*. Mol. Gen. Genomics. 291, 1545–1559. doi: 10.1007/s00438-016-1203-2, PMID: 27033553

[ref64] Wang Y. Wang J. C. Guo D. D. Zhang H. B. Che Y. H. Li Y. Y.. (2021a). Physiological and comparative transcriptome analysis of leaf response and physiological adaption to saline alkali stress across pH values in alfalfa *(Medicago sativa)*. Plant Physiol. Biochem. 167, 140–152. doi: 10.1016/j.plaphy.2021.07.040, PMID: 34352517

[ref65] Wang Y. Yu Y. T. Zhang H. B. Huo Y. Z. Liu X. Q. Che Y. H.. (2021b). The phytotoxicity of exposure to two polybrominated diphenyl ethers (BDE47 and BDE209) on photosynthesis and the response of the hormone signaling and ROS scavenging system in tobacco leaves. J. Hazard. Mater. 426:128012. doi: 10.1016/j.jhazmat.2021.128012 34923383

[ref66] Wu Y. Wen J. Xia Y. Zhang L. Du H. (2022). Author notes evolution and functional diversification of R2R3-MYB transcription factors in plants. Hortic. Res. 9:uhac058. doi: 10.1093/hr/uhac058, PMID: 35591925 PMC9113232

[ref67] Xie Z. Li D. Wang L. Sack F. D. Grotewold E. (2010). Role of the stomatal development regulators FLP/MYB88 in abiotic stress responses. Plant J. 64, 731–739. doi: 10.1111/j.1365-313X.2010.04364.x, PMID: 21105921

[ref68] Xu R. Wang Y. Zheng H. Lu W. Wu C. Huang J.. (2015). Salt-induced transcription factor MYB74 is regulated by the RNA-directed DNA methylation pathway in Arabidopsis. J. Exp. Bot. 66, 5997–6008. doi: 10.1093/jxb/erv312, PMID: 26139822 PMC4566987

[ref69] Yan K. He W. Bian L. Zhang Z. Tang X. An M.. (2020). Salt adaptability in a halophytic soybean (*Glycine soja*) involves photosystems coordination. BMC Plant Biol. 20:155. doi: 10.1186/s12870-020-02371-x, PMID: 32276592 PMC7149873

[ref70] Yang J. Y. Zheng W. Tian Y. Wu Y. Zhou D. W. (2011). Effects of various mixed salt-alkaline stresses on growth, photosynthesis, and photosynthetic pigment concentrations of *Medicago ruthenica* seedlings. Photosynthetica 49, 275–284. doi: 10.1007/s11099-011-0037-8

[ref71] Yu Y. T. Wu Z. Lu K. Bi C. Liang S. Wang X. F.. (2016a). Overexpression of the MYB37 transcription factor enhances abscisic acid sensitivity, and improves both drought tolerance and seed productivity in *Arabidopsis thaliana*. Plant Mol. Biol. 90, 267–279. doi: 10.1007/s11103-015-0411-1, PMID: 26646286 PMC4717180

[ref72] Yu Y. T. Wu Z. Lu K. Bi C. Liang S. Wang X. F.. (2016b). Overexpression of the MYB transcription factor MYB28 or MYB99 confers hypersensitivity to abscisic acid in arabidopsis. J. Plant Biol. 59, 152–161. doi: 10.1007/s12374-016-0463-z

[ref73] Zhang C. G. Leung K. K. Wong Y. S. Tam N. F. Y. (2006). Germination, growth and physiological responses of mangrove plant (*Bruguiera gymnorrhiza*) to lubricating oil pollution. Environ. Exp. Bot. 60, 127–136. doi: 10.1016/j.envexpbot.2006.09.002

[ref74] Zhang H. H. Li X. Xu Z. S. Wang Y. Teng Z. Y. An M. J.. (2020a). Toxic effects of heavy metals Pb and Cd on mulberry (*Morus alba L.*) seedling leaves: photosynthetic function and reactive oxygen species (ROS) metabolism responses. Ecotoxicol. Environ. Saf. 195:110469. doi: 10.1016/j.ecoenv.2020.110469 32179235

[ref75] Zhang H. H. Li X. Zhang S. B. Yin Z. P. Zhu W. X. Li J. B.. (2018a). Rootstock alleviates salt stress in grafted mulberry seedlings: physiological and PSII function responses. Front. Plant Sci. 9:1806. doi: 10.3389/fpls.2018.01806, PMID: 30619391 PMC6297837

[ref720] Zhang H. H. Shi G. L. Shao J. Y. Li X. Li M. B. Meng L.. (2019). Photochemistry and proteomics of mulberry (*Morus alba* L.) seedlings under NaCl and NaHCO_3_ stress. Ecotoxicol. Environ. Saf. 184:109624. 31487570 10.1016/j.ecoenv.2019.109624

[ref76] Zhang H. H. Wang Y. Li X. He G. Q. Che Y. H. Teng Z. Y.. (2020b). Chlorophyll synthesis and the photoprotective mechanism in leaves of mulberry (*Morus alba* L.) seedlings under NaCl and NaHCO_3_ stress revealed by TMT-based proteomics analyses. Ecotoxicol. Environ. Saf. 190:110164. doi: 10.1016/j.ecoenv.2020.110164 31927191

[ref77] Zhang P. Wang R. Yang X. Ju Q. Li W. Lü S.. (2020e). The R2R3-MYB transcription factor AtMYB49 modulates salt tolerance in Arabidopsis by modulating the cuticle formation and antioxidant defence. Plant Cell Environ. 43, 1925–1943. doi: 10.1111/pce.13784, PMID: 32406163

[ref78] Zhang H. H. Xu Z. S. Guo K. W. Huo Y. Z. He G. Q. Sun H. W.. (2020d). Toxic effects of heavy metal cd and Zn on chlorophyll, carotenoid metabolism and photosynthetic function in tobacco leaves revealed by physiological and proteomics analysis. Ecotoxicol. Environ. Saf. 202:110856. doi: 10.1016/j.ecoenv.2020.110856, PMID: 32629202

[ref79] Zhang H. H. Xu Z. S. Huo Y. Z. Guo K. W. Wang Y. He G. Q.. (2020c). Overexpression of Trx CDSP32 gene promotes chlorophyll synthesis and photosynthetic electron transfer and alleviates cadmium-induced photoinhibition of PSII and PSI in tobacco leaves. J. Hazard. Mater. 398:122899. doi: 10.1016/j.jhazmat.2020.122899, PMID: 32450465

[ref80] Zhang H. H. Xu N. Wu X. Y. Wang J. R. Ma S. L. Li X.. (2018b). Effects of four types of sodium salt stress on plant growth and photosynthetic apparatus in sorghum leaves. J. Plant Interact. 13, 506–513. doi: 10.1080/17429145.2018.1526978

[ref670] Zhang H. H. Zhong H. X. Wang J. F. Sui X. Xu N. (2016). Adaptive changes in chlorophyll content and photosynthetic features to low light in *Physocarpus amurensis* Maxim and *Physocarpus opulifolius* “Diabolo”. Peer J. 4:e2125., PMID: 27366639 10.7717/peerj.2125 PMC4924129

[ref81] Zhao Y. Yang Z. Ding Y. Liu L. Han X. Zhan J.. (2019). Overexpression of an R2R3 MYB gene, GhMYB73, increases tolerance to salt stress in transgenic Arabidopsis. Plant Sci. 286, 28–36. doi: 10.1016/j.plantsci.2019.05.021, PMID: 31300139

[ref82] Zheng X. Li H. Chen M. Zhang J. Tan R. Zhao S.. (2020). *Smi-miR396b* targeted SmGRFs, SmHDT1, and SmMYB37/4 synergistically regulates cell growth and active ingredient accumulation in Salvia miltiorrhiza hairy roots. Plant Cell Rep. 39, 1263–1283. doi: 10.1007/s00299-020-02562-8 32607753

[ref83] Zhou C. Li C. (2016). A novel R2R3-MYB transcription factor BpMYB106 of birch (*Betula platyphylla*) confers increased photosynthesis and growth rate through up-regulating photosynthetic gene expression. Front. Plant Sci. 7:315. doi: 10.3389/fpls.2016.00315 27047502 PMC4801893

[ref84] Zhu J. K. (2002). Salt and drought stress signal transduction in plants. Annu. Rev. Plant Biol. 53, 247–273. doi: 10.1146/annurev.arplant.53.091401.143329, PMID: 12221975 PMC3128348

[ref85] Zhuang W. W. Li J. Cao M. H. Feng W. J. Li Y. P. (2010). Changes of osmotic adjusting substances in leaves of ammodendron argenteum seedlings under salt and drought stress. Acta Botan. Boreali-Occiden. Sin. 30, 2010–2015.

